# Book Reviews

**Published:** 1895-10

**Authors:** 


### Book Reviews.

A SYSTEM OF SURGERY. By American authors. Edited by FREDERIC S. DENNIS, M. D., Professor of the Principles and Practice of Surgery. Bellevue Hospital Medical College, New York; President of the American Surgical Association, etc., assisted by JOHN S. BILLINGS, M. D., LL. D., D. C. L., Deputy Surgeon-General, U. S. A. To be completed in four imperial octavo volumes, containing about 900 pages, each with index. Volume II., 915 pages, 515 engravings and ten colored plates. Price, per volume, $6.00 in cloth; $7.00 in leather; $8.50 in half morocco, gilt back and top. Philadelphia: Lea Brothers & Co. 1895.

This volume opens with a section on minor surgery, consisting of 136 pages, written by Henry R. Wharton. It gives minute details for bandaging, with copious illustrations, most of which are the author's own, from photographs of applied bandages. The fifty-six pages devoted to this part of the subject approach nearly to a clinic. Then follows instructions for the application of fixed dressings, such as plaster-of-Paris to fractures and to spinal curvatures. A short account of materials used in surgical dressings is given, among which are instructions for strapping organs in different parts of the body, irrigation, use of moxa, introduction of setons, cautery, scarification, transfusion, employment of artificial respiration, aspiration, use of the stomach pump, hypodermic injections and the rectal tube. Electro-therapy is touched upon, instructions given for the employment of the cystoscope and similar instruments, and the introduction of catheters and bougies. Wharton is very unsatisfactory in his directions about washing out the bladder. He omits mention of the method employed by Daggett, of Buffalo, which is, by far, the best yet devised, and is a distinct addition to the therapy of cystic disease. Surgical needles, sutures and ligatures are well described ; so, too, is acupressure. This is practically a republication of the author's treatise on minor surgery which is already in the hands of the profession, but to which some additions have been made.

Plastic surgery forms the subject of the next section, which is written by George R. Fowler, and covers fifty pages of the book. Hare-lip and cleft-palate command considerable attention, with copious illustrations. So, too, with regard to rhinoplasty. Bone-grafting might well receive more detailed treatment ; but, on the whole, this section on plastic surgery is very satisfactory.

The third section is devoted to a consideration of military surgery and the care of the wounded on the battlefield, and is written by Lieutenant-Colonel W. H. Forwood, U. S. army. The author gives an account of the immediate care of wounded and their transportation. He writes an interesting article on the medical officer and his work, and relates a great truth in the following words : "After every great battle, all the more severely wounded ought to remain and be taken care of near as possible to where their wounds were received."

A section on diseases of the bones next follows, from the pen of Nicholas Senn. This is a most important subject, one to which insufficient attention is paid, but of which this author is a master. Into the forty-six pages here presented he has condensed information of great value.

Orthopedic surgery is next presented, by Virgil P. Gibney, than whom no one is more competent to speak authoritatively on this subject. When a man cures a deformed human being, or so modifies his crippled condition as to make him self-supporting, he has performed a most important service to the community. There is no department of surgery that bears so close a relation to political economy as this. Gibney is very strong in his management of hip-disease. The whole section is intensely interesting and well illustrated.

Next follows a section devoted to aneurism, written by Lewis A. Stimson, which is, by far, the amplest and best setting forth of the subject we have seen. Its pathology is clear and its treatment is intelligent. Surgery of the artery and veins is given by Frederic S. Dennis, and surgery of the lymphatic system by Frederic Henry Gerrish.

The most voluminous section in the book, consisting of 285 pages, is devoted to the consideration of diseases and injuries of the head, and is written by Roswell Park. Brain surgery has attracted great attention of late, and advances in this department have been most marked. Cerebral localisation has contributed to aid the surgeon, who, in turn, has relieved many diseases heretofore thought to be incurable, not to say inoperable. Intra-cranial tumors are now diagnosticated and successfully excised that formerly were allowed to progress to fatal issue. This is one of the most satisfactory dissertations on the subject of head injuries and diseases that has yet appeared, and it is well worth the careful study of every surgeon as well as neurologist. The author ought to be induced to put it forth as a separate treatise.

Closely allied to the foregoing is the surgery of the spine, hence it is fit that it should follow it in close order. This section is written by W. W. Keen, and is both comprehensive and scholarly. It has, too, surrounding it the atmosphere of the practical surgeon, which lends interest to its instructive pages.

Finally, we find surgery of the nerves described by John B. Roberts in an admirable essay of fifty pages.

This volume is full of interest from cover to cover, and a detailed examination of it fully justifies the opinion we have previously expressed of the work.

THE DEFORMITIES OF THE HUMAN FOOT, WITH THEIR TREATMENT. By W. J. WALSHAM, M. B., C. M., Aberd.; F. R. C. S., Eng. ; Senior Assistant Surgeon, Surgeon in Charge of the Orthopedic Department and Lecturer in Anatomy, St. Bartholomew's Hospital, etc., and William Kent Hughes, M. B., Lond. ; M. B., Melb. ; M. R. C. S., Eng. ; L. R. C. P., Lond. ; Orthopedic Surgeon, St. Vincent's Hospital ; Assistant Surgeon, Children's Hospital, Melbourne ; Surgical Tutor, Trinity College, Melbourne, etc. Extra muslin ; 8vo, pp. 550. Price, $4.50. New York : William Wood & Company. 1895.

The most interesting branch of orthopedic surgery is that relating to deformities of the feet, and in no department of surgery has there been more improvement within the last twenty-five years than in this. During the plastic period of infancy and childhood, deformities can be corrected by the knife or other mechanical means, that otherwise would cripple the individual for life. To cure the deformed and make the dependent self-supporting is among the highest functions of the surgeon.

The authors of this work are distinguished for their skill and have recorded in it their experience during thirteen years' service in the orthopedic department of St. Bartholomew's hospital. It will readily be seen that such a work must be of the most practical character, written, as it is, by practical men and based on their own clinical experience, and it will be easily taken as authority on the subject. The book is beautifully printed, profusely and excellently illustrated. It should find its place on the book shelves of the practiser of general medicine.

---

THE CARE OF THE BABY. A Manual for Mothers and Nurses, containing practical directions for the management of infancy and childhood in health and disease. By J. P. CROZER GRIFFITH, M. D., Clinical Professor of Diseases of Children in the Hospital of the University of Pennsylvania ; Professor of Clinical Medicine in the Philadelphia Polyclinic and School for Graduates in Medicine ; Physician to the Children's Hospital, etc. Small 8vo, pp. 392. Price, $1.50. Philadelphia : W. B. Saunders, 925 Walnut street. 1895.

This book is by an experienced author and he has told many valuable things in it that ought to be known by mothers and nurses as well as those about to become mothers. In it he discourses on the hygiene of pregnancy, the characteristics of a healthy baby, the growth of its mind and body, the methods of bathing, dressing and feeding children of different ages, the hours for sleeping, physical and mental exercise and training and the proper qualities of children's nurses and rooms.

Nearly one-half of the book is devoted to the consideration of the sick baby, while an appendix is added in which dietary and remedies are formulated. The whole is written in a simple and pleasant manner, without marked technicalities, but yet with scientific accuracy.

This is one of the books that will do good in the hands of mothers and nurses, for whom it is specially intended.

REMOTE CONSEQUENCES OF INJURIES OF THE NERVES AND THEIR TREATMENT. An examination of the present condition of wounds received in 1863–65, with additional illustrative cases. By JOHN K. MITCHELL, M. D., Assistant Physician to the Orthopedic Hospital and Infirmary for Nervous Diseases, Philadelphia ; Lecturer on Physical Diagnosis in the University of Pennsylvania. Duodecimo, pp. 233, with twelve illustrations. Cloth, $1.75. Philadelphia: Lea Brothers & Co. 1895.

This author has been delving in a new field, or, perhaps, more properly, has been cultivating an old field in a new manner. The importance of a more thorough understanding of nerve injuries by surgeons and neurologists can scarcely be overestimated. The records of the surgeon-general's office at Washington are rich with important examples of gun-shot injuries to nerves. Dr. Mitchell has taken great pains to trace a large number of these to their remote results and has recorded the information obtained in this book. The difficulties incurred in this research have been many, owing to changes of address, fear of losing their pension and other causes, known and unknown, that have entered into the search for many old soldiers whose cases possessed interest. The pension office lent valuable aid, without which, in many instances, it would have been impossible to succeed.

We commend the study of this book to all interested in the subject.

---

FORMULAIRE DES SPECIALITES PHARMACEUTIQUES, composition, indications thèrapeutiques, mode d'emploi et dosage, á l'usage des mèdecins, par le D. M. GAUTIER, ancien interne des hôpitaux, et F. RENAULT, pharmacien de 1re classe, lauréat de l'École de pharmacie. 1 vol. in-18 de 300 pages, cartonné 3 fr. Librairie J.-B. Baillière et Fils, 19 rue Hautefeuille (près du boulevard Saint-Germain), á Paris. 1895.

This is a very conveniently arranged little book and will be found useful in refreshing the memory concerning the composition and uses of special pharmaceutical preparations. It furnishes the latest information concerning the use of most such and is preceded by a commentary of Professor Cornil on his personal experience with these preparations.

---

A GUIDE TO THE ANTISEPTIC TREATMENT OF WOUNDS. By Dr. C. SCHIMMELBUSCH, Assistant in the Royal Surgical Clinic of the University of Berlin. Preface by Prof. E. Von Bergmann. Translated from the second revised German edition, with express permission of the author, by Frank J. Thornbury, M. D., Lecturer on Bacteriology, University of Buffalo, N. Y., etc. Octavo, pp. xii.—233, with forty-three illustrations. New York : G. P. Putnam's Sons, 27 West Twenty-third street. 1895.

This book contains an excellent setting forth of the principles that should govern the aseptic treatment of wounds. It should be the aim to treat all wounds aseptically, and students and physicians need the most thorough training in regard to personal cleanliness as well as in the careful and clean manipulation of wounds and dressings.

The author teaches these principles in the most systematic and simple manner, and the translator has performed his part with ability such as to receive the encomiums of the author.

The book is well illustrated and handsomely printed.

---

TRANSACTIONS OF THE MEDICAL ASSOCIATION OF CENTRAL NEW YORK. Twenty-seventh Annual Meeting, Buffalo, N. Y., October 16, 1894. BUFFALO MEDICAL JOURNAL Print. 1895.

This brochure of eighty-four pages contains the proceedings of, and papers read before this, association at its last annual meeting. These papers have appeared in the JOURNAL, so that most of our readers are familiar with them.

It is an excellent plan for every medical society to print and preserve its work in an accessible form for reference. If this association had done so at the outset, its members could now enjoy a valuable collection of medical literature. When a society fails to preserve its work, it does itself injustice, and it is to be hoped that this excellent association will continue the method so auspiciously begun.

---

SEVENTEENTH ANNUAL REPORT OF THE STATE BOARD OF HEALTH OF ILLINOIS. Being for the year ended December 31, 1894. With an appendix containing the official register of physicians and midwives, 1895. Springfield: Ed. F. Hartman, State Printer. 1895.

A large part of this volume is given up to the publication of the official register of physicians in the state of Illinois. The reduction in the appropriation for publication has rendered it necessary to condense much of this material and to omit sundry data that heretofore have been published. The good work of this board, inaugurated by the late Dr. John H. Rauch, in improving the standard of medical education, has been the subject of favorable comment all over the land.

---

THE AMERICAN ACADEMY OF RAILWAY SURGEONS. Official report of first meeting held at Chicago, Ill., November 9-10, 1895. Edited by R. Harvey Reed, M. D., Columbus, O. Chicago: American Medical Association Press. 1895.

This little volume contains the proceedings and papers read, together with discussions thereon, at the first meeting of this new organization. It will prove of interest not only to its members but many others engaged in the practice of railway surgery.

THE POCKET MATERIA MEDICA AND THERAPEUTICS. A Résumé of the Action and Doses of all Officinal and Non-officinal Drugs now in Common Use. By C. HENRI LEONARD, A. M., M. D., Professor of the Medical and Surgical Diseases of Women and Clinical Gynecology in the Detroit College of Medicine, etc. Second edition, revised and enlarged. Cloth, large 16mo, 367 pages. Price, post-paid, $1.00. Detroit: The Illustrated Medical Journal Co., Publishers. 1895.

In this second edition of Leonard's popular work sixty-seven pages have been added, a new and complete cross-index has been prepared, typographical errors corrected and in other ways it has been greatly improved and brought forward to the present period. The descriptive arrangement of the drugs is as follows: Alphabetically the drug, with its pronunciation, (officinal or non-officinal standing indicated,) genitive case-ending, common name, dose and metric dose. Then the English, French and German synonyms. If a plant, the part used, habitat, natural order, botanic description, with alkaloids, if any; if a mineral, its chemical symbol, atomic weight, looks, taste, how found, its peculiarities. Then the action and uses of the drug or compound, its antagonists, its incompatibles, its synergists and then antidotes. Then follow its officinal and non-officinal preparations with their medium and maximum doses. Altogether it is a handy volume for physician, druggist or student, and will be frequently appealed to if in one's possession.

---

EXERCISE AND FOOD FOR PULMONARY INVALIDS. By CHARLES DENISON, A. M., M. D., Denver, Col., Professor of Diseases of the Chest and of Climatology, University of Denver, etc. Price, 35 cents. Denver: The Chain & Hardy Company. 1895.

This monograph is written by an author who has experience on the subject and it will be found to contain many points of interest to those afflicted with pulmonary disease. One of its special purposes, however, seems to be to advertise the instruments that have been devised by the author.

